# Case report: the management for a gestational hypertensive woman with influenza A virus pneumonia and peripartum cardiomyopathy

**DOI:** 10.1186/s12884-022-04814-9

**Published:** 2022-06-17

**Authors:** Kwok-On Ng, Lok-Hi Chow, Chun-Chang Yeh, Eagle Yi-Kung Huang, Wei-Cheng Liu, Ping-Heng Tan

**Affiliations:** 1 Department of Anesthesiology, Ditmanson Medical Foundation Chia-Yi Christian Hospital, Chia Yi, Taiwan; 2 Department of Anesthesiology, School of Medicine, Taipei Veterans General Hospital, and National Yang-Ming Chiao-Tung University, Taipei, Taiwan; 3 Department of Anesthesiology of Tri-Service General Hospital &, National Defense Medical Center, Taipei, Taiwan; 4 Department of Pharmacology, National Defense Medical Center, Taipei, Taiwan; 5 Department of Anesthesiology, Chi Mei Medical Center, Tainan, Taiwan

**Keywords:** Peripartum cardiomyopathy, Caesarean section, Pneumonia, influenza A

## Abstract

**Background:**

Peripartum cardiomyopathy (PPCM) is defined as an idiopathic cardiomyopathy occurring in the last month of pregnancy or the first 6 months postpartum without an identifiable cause. PPCM is suspected to be triggered by the generation of a cardiotoxic fragment of prolactin and the secretion of a potent antiangiogenic protein from the placental, but no single factor has been identified or defined as the underlying cause of the disease. Influenza virus can cause PPCM through immune-mediated response induced by proinflammatory cytokines from host immunity and endothelial cell dysfunction. We report a case in a parturient woman undergoing a cesarean delivery, who had influenza A pneumonia and PPCM.

**Case presentation:**

A parturient woman at 40 weeks and 1 day of gestation who had experienced gestational hypertension accompanied by pulmonary edema developed hypotension after undergoing an emergency cesarean delivery. An elevation of N-terminal prohormone of brain natriuretic peptide (NT-proBNP) was noted, and echocardiography revealed a left ventricular ejection fraction of 20%. She underwent a nasopharyngeal swab test, in which influenza A antigen was positive. She was diagnosed as having PPCM and received anti-viral treatment. After antiviral treatment, hemodynamic dysfunction stabilized. We present and discuss the details of this event.

**Conclusion:**

PPCM is a heart disease that is often overlooked by medical personnel. Rapid swab tests, serum creatine kinase measurement, and echocardiography are imperative diagnostic approaches for the timely recognition of virus-associated cardiomyopathy in peripartum women with influenza-like disease and worsening dyspnea, especially during the epidemic season. Prompt antiviral treatment should be considered, particularly after PPCM is diagnosed.

**Supplementary information:**

The online version contains supplementary material available at 10.1186/s12884-022-04814-9.

## Background

Peripartum cardiomyopathy (PPCM), a rare condition, is defined as an idiopathic cardiomyopathy that occurs in the last month of pregnancy or the first 6 months postpartum without an identifiable cause [1]. Because of the lack of consensus on diagnostic criteria, PPCM is only diagnosed by exclusion. In the United States, the estimated incidence of PPCM is 1:900 to 1:4000 live births [2]. Most women develop PPCM postpartum, mainly in the first month after delivery [3]. The prognosis of PPCM appears to vary geographically and socioeconomically, with a reduced likelihood of recovery for people of black ancestry in North America [4]. More than half of affected patients recover systolic function, while the others have persistent cardiac dysfunction [5]. A left ventricular ejection fraction (LVEF) of < 30% and a left ventricular end-diastolic diameter of ≥ 6.0 cm is indicative of a significantly decreased likelihood of left ventricular recovery [4]. PPCM is suspected to be triggered by the generation of a cardiotoxic prolactin fragment and the secretion of a potent antiangiogenic protein from the placenta [6], but no single factor has been identified or defined as the underlying cause of the disease.

The development of myocarditis has been reported in up to 20% of hospitalized patients with alleged severe influenza [7–9], which could be confirmed by conducting cardiac magnetic resonance (CMR) imaging to identify considerable intramyocardial inflammation and endomyocardial tissue biopsy to detect viral genomes [10]. Influenza can cause PPCM through an immune-mediated response induced by proinflammatory cytokines involved in host immunity and endothelial cell dysfunction [11]. The clinical presentation of PPCM resembles myocarditis. Myocarditis diagnosed through CMR imaging or endomyocardial tissue biopsy must be excluded for the definite diagnosis of PPCM.

### Case presentation

**Fig. 1:**
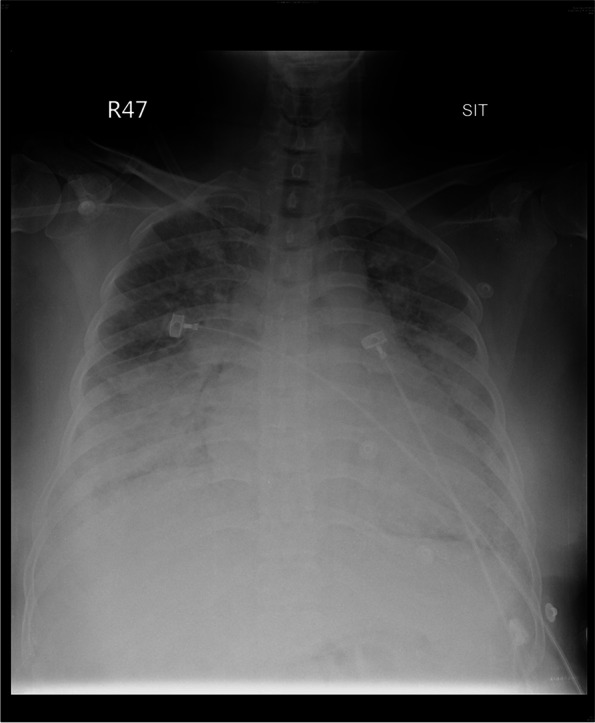
Chest radiography revealed cardiomegaly, bilateral interstitial infiltrates, and patchy opacities (multifocal consolidations)

A 37-year-old woman (gravida 7, para 4) at 40 weeks and 1 day of gestation was referred from a maternity clinic to our emergency department (ED) because of progressive dyspnea. She weighed 74 kg and was 160 cm tall, with no systemic disease including cardiovascular disease, previous medical or surgical diseases of note. The patient reported experiencing muscle aches and fatigue for a week, which she initially attributed to pregnancy. Three days prior to ED admission, she developed a dry cough, shortness of breath and orthopnea that persisted to admission. In the ED, her blood pressure, pulse rate, and respiratory rate were 162/118 mmHg, 84 beats/minute, and 26 breaths/minute, respectively. Intravenous trandate 5 mg was administered to control blood pressure. The body temperature was 37.2 °C. Despite the administration of supplemental oxygen (5 L/min) via a nasal cannula, her pulse oxygen saturation level remained at 91%, with a high of 93%. Examination revealed tachypnea, an appearance of breathlessness while lying flat, and systemic edema in the feet and ankle in particular. Coarse crackles breathing sounds were heard from both lungs on auscultation. Biochemical tests were unremarkable (Alanine transaminase: 10 IU/L, Aspartate transaminase: 18 IU/L, Prothrombin time: 10.5 secs, Activated partial thromboplastin time: 28 secs, Blood urea nitrogen: 10.2 mg/dL, Creatinine: 0.41 mg/dL) and absent of proteinuria in a urine test. Blood data showed a mildly elevated white cell count (9,400 /µL), while C-reactive protein was 0.746 mg/dL (normal < 0.5 mg/dL). Electrocardiography (ECG) showed sinus tachycardia with a nonspecific ST-T segment. Chest radiography revealed cardiomegaly, bilateral interstitial infiltrates, and patchy opacities (multifocal consolidations) consistent with pulmonary edema (Fig. 1). She followed our advice of an emergency cesarean delivery, which was given out of concern for her deteriorating condition and the futility of planning a vaginal birth for a woman of nearly postterm pregnancy.

**Fig. 2:**
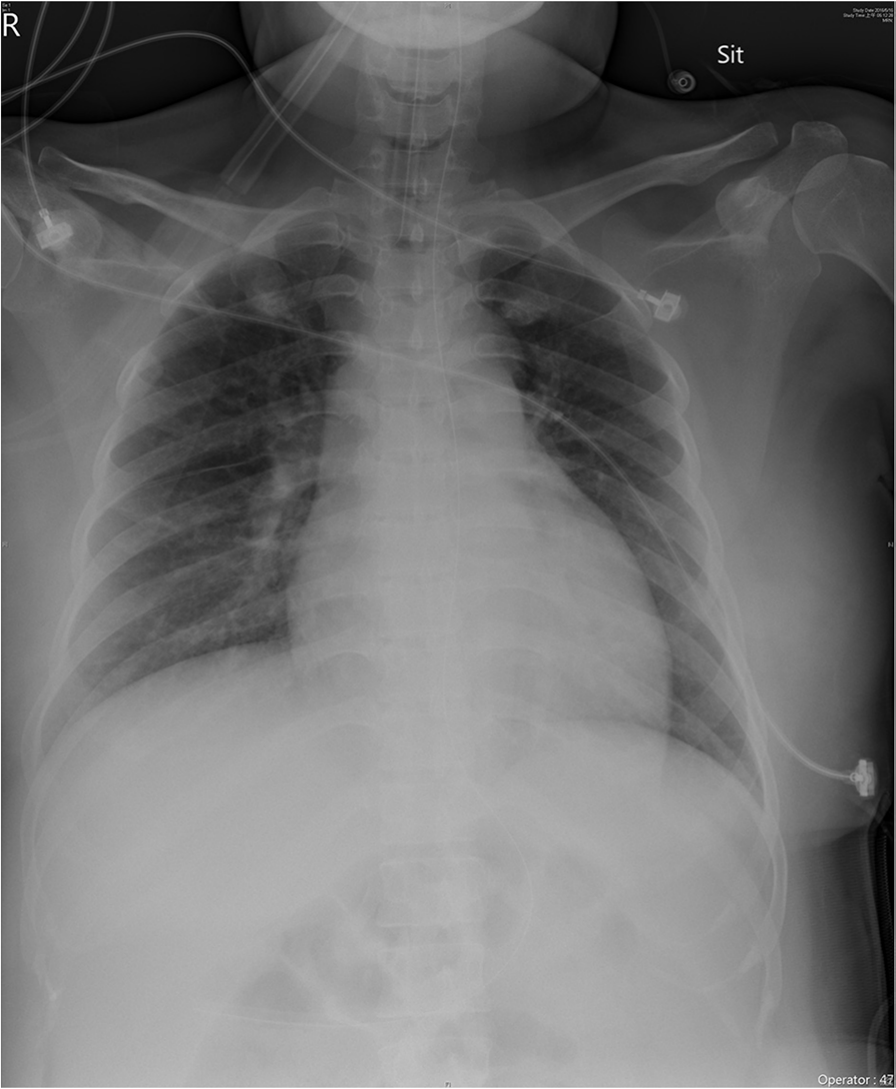
Chest radiograph revealed mild bilateral infiltration

In the operating room, her vital signs were a blood pressure of 160/100 mmHg, pulse rate of 90, respiratory rate of 28, and pulse oxygen saturation of 97% while using an oxygenated facial mask. General anesthesia was induced with propofol (150 mg) and succinylcholine (100 mg) intravenously to facilitate intubation. She was intubated with a Mallinckrodt tube with a 7.0-mm internal diameter. The operation proceeded uneventfully. Over the 65 min operative period, a total of 300 mL of normal saline were administered intraoperatively, and 70 mL of urine were excreted. The male newborn with a weight of 4,048 g and Apgar scores of 8 and 9 at 1 and 5 min, respectively, was immediately admitted to the neonatal intensive care unit (ICU) for observation. Subsequently, the patient was transferred to the ICU, where she had to be placed on a ventilator with a pressure support mode of 15 cmH_2_0, inspiratory oxygen fraction of 60%, and positive end-expiratory pressure of 5 cmH_2_O. Pulse oximetry value was 95%, and blood pressure was 150/90 mmHg. Laboratory data obtained at the 8th hour post operation were Alanine transaminase: 33 IU/L, Aspartate transaminase: 30 IU/L, Blood urea nitrogen: 14.2 mg/dL, Creatinine: 0.64 mg/d, WBC: 13,560 /µL and Hgb: 11 g/dL. For every 4 h during the initial intensive care, the blood pressure was 140/90, 130/88/, 120/70 mmHg, respectively. Fourteen hours postoperatively (on postpartum day 1), her blood pressure decreased to 90/60 mmHg in the absence of antihypertensives. ECG monitoring detected sinus tachycardia with a rate of 110–130 beats/min and frequent ventricular premature complexes. Her urine output decreased from 80 to 15 mL/h. Cardiac enzymes were collected and revealed a troponin I level of 0.687 ug/mL (normal < 0.016 ug/mL), creatinine phosphokinase level of 423 units/L (normal < 170 units/L), and creatinine kinase myocardial band level of 5.5 ng/mL (normal < 6.6 ng/mL). The assessed plasma brain-type natriuretic peptide level was 276 pg/mL (normal < 116 pg/mL). Bedside echocardiography revealed a dilated left ventricular chamber with eccentric hypertrophy and severely reduced left ventricular motion with an estimated ejection fraction of 20%. Blood data showed obvious leukocytosis (14,000/mm^3^; neutrophils, 85.5%) and elevated C-reactive protein (7.8 mg/L). Despite the patient being started on medical supportive treatment that included inotropes and diuretics, her hemodynamic dysfunction continued. Given her history of influenza-like symptoms, she underwent a nasopharyngeal swab test, in which she was positive for influenza A antigen. She was diagnosed as having PPCM, and we administered an antiviral agent (oseltamivir) at a dose of 0.12 g/kg/day for 3 days from postpartum day 2. This treatment stabilized her clinical condition. On postpartum day 4, chest radiography revealed reduced pulmonary edema with mild bilateral pulmonary infiltration (Fig. 2), and she was weaned off the ventilator and extubated. On the same day, the result of laboratory tests mitigated, revealing a troponin I level of 0.337 ug/mL, creatinine phosphokinase level of 257 units/L, and creatinine kinase myocardial band level of 2.5 units/L. Owing to the improvement of hemodynamic parameters on postpartum day 5, we discontinued the inotrope therapy and transferred her to the general ward with oral enalapril 1.25 mg/day, furosemide 20 mg/day, and spironolactone 25 mg/day. Following a steady improvement in her daily activity levels, she was discharged on postpartum day 7 with symptoms corresponding to New York Heart Association Functional Classification Class II and received follow-up appointments with a cardiologist. Two years after discharge from the hospital, repeat echocardiography showed that her LVEF had recovered to 52%.

## Discussion and conclusions

Gestational hypertension, which was considered a risk factor for PPCM, occurs in up to 10% of pregnancies worldwide and accounts for 20% of all PPCM cases [12]. Kamiya et al. surveyed 102 patients with PPCM complicated with and without hypertensive disorders and concluded that hypertension in pregnancy was not causative in the development of PPCM [13]. Pregnant women with comorbidities are particularly prone to dangerous complications. Influenza A antibody was detected in our patient, in addition to increased N-terminal prohormone of brain natriuretic peptide levels, indicative of heart failure [14]. Exclusion of influenza-related acute myocarditis was required. However, endomyocardial biopsy and magnetic resonance imaging are not imperative nor usual tools for the clinical diagnosis of myocarditis in our hospital. The serum creatinine kinase and troponin levels in the patient were increased, but increased creatinine kinase levels in the absence of myocardial injury during peripartum [15] and increased troponin levels during the acute stage of PPCM are reported in the literature [16]. Furthermore, her creatinine kinase myocardial band level did not exceed the threshold for the diagnosis of myocarditis. Ntusi et al. conducted a case-comparison study of PPCM and hypertensive heart failure of pregnancy (HHFP) and found a lower LVEF (23.8 ± 8.3% vs 49.9 ± 18.7%) and an increased LV dimension in the diastole (> 5.5 cm) in the PPCM group compared with the HHFP group [17]. The EF of 20% and LV dimension of 6.01 cm in diastole of our case are consistent with the features of PPCM mentioned by Ntusi et al.

Several cases of influenza-related PPCM are reported in the literature. Muroya et al. described a pregnant woman with preeclampsia who developed heart failure complicated with pulmonary edema on day 4 postpartum. Owing to an elevated white blood cell count, antibody titers to influenza virus, and a negative inflammatory finding in endomyocardial tissue, the patient was diagnosed as having PPCM instead of myocarditis. After intravenous immunoglobulin administration, her cardiac function improved with the nullification of the antibody titer [18]. Chan et al. reported cases of two pregnant women with PPCM. The patients had H1N1 viral pneumonia, developed acute respiratory distress syndrome, and finally underwent emergency cesarean delivery [19]. Both women developed heart failure postpartum, but the levels of troponin and creatinine kinase myocardial enzymes were within the normal range. They were administered oseltamivir, a neuraminidase inhibitor. One woman recovered with persistent cardiomyopathy; the other recovered without any long-term consequences. Chan et al. argued that prompt antiviral treatment could improve survival, especially in pregnant women with influenza-related heart failure. Suárez et al. recently reported the case of a pregnant patient with H1N1 influenza viral infection who developed heart failure after the delivery of twins a week prior [20]. H1N1 myocarditis was disapproved by magnetic resonance imaging results, and the diagnosis of PPCM was preferred. The patient was initially treated with conventional congestive heart failure therapy. Her condition did not improve until she was treated with oseltamivir. They concluded that oseltamivir, which is recommended by the Japanese Association of Infection for all patients with influenza [21], is an effective treatment for influenza-triggered PPCM. These promising signs set a precedent for the management of future cases. Further research is required to understand whether the mechanisms of treatments for influenza-related myocarditis and PPCM are the same. In this case, the patient’s hemodynamic dysfunction stabilized within 2 days of starting oseltamivir. This indicates that oseltamivir is likely a viable option for treating influenza-related cardiomyopathy in the future.

Prompt initiation of antiviral treatment in patients with suspected or confirmed influenza is paramount and is not contraindicated by a negative rapid influenza test [22, 23]. In a case series of maternal exposure to oseltamivir, no evidence suggested that maternal exposure to oseltamivir was associated with adverse pregnancy or fetal outcomes [24].

Testing for influenza in pregnant women is dependent on symptoms. Differentiating the subtle symptoms of heart failure (exertion dyspnea, fatigue, and pedal edema) from normal observations made during late pregnancy is difficult for some clinicians. Physicians often misinterpret the symptoms of pregnant women falling sick with influenza as gravidity. In a study of patients with cardiomyopathy of unknown cause occurring during pregnancy, Goland et al. observed that 48% had a delayed diagnosis, which postpones medical intervention and results in much more severe illness [25]. Echocardiography is crucial for a peripartum woman with symptoms and signs of cardiac failure because it helps to establish the diagnosis, adjust the drug dosage, and obtain prognostic information. Furthermore, the incidence of malignant arrhythmia and sudden cardiac death increases if LVEF is < 30% [26].

The principles for managing acute heart failure resulting from PPCM are similar to those for managing any other type of acute heart failure [27], with special attention paid to avoiding drugs with adverse fetal effects in women who are still pregnant. Angiotensin-converting enzyme inhibitors, angiotensin receptor blockers, and mineralocorticoid receptor antagonists are contraindicated during pregnancy. Although prolactin plays a role in the pathogenesis of PPCM, the use of bromocriptine for treatment requires further investigation with a clinical trial.

Pregnant women are advised to get vaccinated during an influenza outbreak. A high vaccination rate prevents both the further spread of the virus and the potential for new dangerous variants to emerge. Inactivated influenza vaccination has been declared safe and effective for pregnant women; hence, vaccination may make the difference between little risk of disease and a risk of dying after contracting the virus. Vaccination also provides a benefit to the newborn, affording a reduced risk of influenza-related pneumonia during the first 6 months after delivery [28, 29].

In conclusion, PPCM is a heart disease that is often overlooked by medical personnel. Rapid swab tests, serum creatine kinase measurement, and echocardiography are imperative diagnostic approaches for the timely recognition of virus-associated cardiomyopathy in peripartum women with influenza-like disease and worsening dyspnea, especially during the epidemic season. Prompt antiviral treatment should be considered, particularly after PPCM is diagnosed.

## Supplementary information

**Additional file 1.** Reply to reviewer 1 and 2 from Rok5bR1.

## Data Availability

All data and materials described in the manuscript will be freely available to any scientist wishing to use them for non-commercial purposes. KON should be contacted if someone wants to request the data.

## Abbreviations

PPCM: Peripartum cardiomyopathy
LVEF: Left ventricular ejection fraction
CMR: Cardiac magnetic resonance
ED: Emergency department
ECG: Electrocardiography
ICU: Intensive care unit
HHFP: Hypertensive heart failure of pregnancy
