# Supplementary material for: Case report: the management for a gestational hypertensive woman with influenza A virus pneumonia and peripartum cardiomyopathy
Source: BMC Pregnancy Childbirth. 2022 Jun 17;22:497. doi: 10.1186/s12884-022-04814-9 (PMC9205026; doi:10.1186/s12884-022-04814-9)
Supplement: Supplementary file 1 — Additional file 1. Reply to reviewer 1 and 2 from Rok5bR1. [file 12884_2022_4814_MOESM1_ESM.doc]

May 26, 2022

Dear Prof. Ashraf Nabhan:

Thanks for very much for your positive decision letter of May 5th, 2022. We also thank the reviewers for their constructive comments, which were very helpful for improving this manuscript.

According to these comments, we provided more data and made several changes in the manuscript.

Please find our point by point responses to all the comments of two reviewers. The corrections in the text are highlighted in yellow.

We hope that with all these changes the manuscript is now suitable for a publication in BMC Pregnancy and Childbirth.

Sincerely yours,

Ping-Heng Tan, MD, PhD

Professor of Anesthesiology

Chi Mei Medical Center

Tainan, Taiwan

**Response Letter (PRCH-D-21-00806)**

**To the Reviewer 1:**

Reviewer 1: This is a case report of a patient who developed a peri-partum cardiomyopathy and not a research paper. I thing it is worth publishing as a reminder of the risks of not recognising the condition. There are some minor grammatical errors, some repetition and the language could be improved. Happy to send my corrections if you wish

*Thank you very much for taking time from your busy schedule to comment on our manuscript. We truly appreciate your kindly suggestions for the grammatical errors, we have corrected some errors, repetition, and language in this revision. Revisions in the text have been highlighted in YELLOW. We are very grateful to you for your comments that help enhance readability of our paper. Thank you.*

**To the Reviewer 2:**

*Thank you very much for taking time from your busy schedule to comment on our manuscript. Our responses to your suggestions and questions are summarized below, with reference to the appropriate pages in the text. Revisions in the text have also been highlighted in YELLOW. We truly appreciate your thoughtful and constructive comments that help make this a better paper. We are very grateful to you for all the comments and insightful suggestions that help enhance both the quality and readability of our paper. Thank you.*

1-Very long title, could be corrected

Response: Thank you for the suggestion. We have revised the title to “The management for a gestational hypertensive woman with influenza A virus pneumonia and peripartum cardiomyopathy”

2- I think history taking lacking some details

Response: The patient was a healthy woman. The history of this patient was not complicated to describe. She had no systemic disease including cardiovascular disease and did not have previous medical history, and attributed muscle aches and fatigue to pregnancy a week before developing dyspnea and admission to hospital.

3-Tempereature not mentioned at all and specially at admission

Response: Thank you for the suggestion. We have revised and mention it in the 2^nd^ paragraph on the page 6

4-Proteinuria test not mentioned at admission

Response: No proteinuria was found in the urine test. We have revised and mention this finding in 5^th^ line on the page 7.

5- Biochemical tests and echo finding at admission should be mentioned in details

Response: Thank you for the suggestion. We have revised and mention it in the 1^st^ paragraph on the page 7, but echo cardiogram was not performed at admission (it is not a routine investigation in ED of our hospital unless it is necessary)

6- clinical signs of heart at admission not mentioned

Response: Thank you for the suggestion. We describe more details of physical examination in the 2^nd^ paragraph on the page 6 and the 1^st^ paragraph on the page 7

7-Antihypertensive was not initiated at operating room or before although blood pressure decreased from 162\118 to 160\100, also O2 saturation increased from at admission 93% to 97% at operating room why?

Response: Thank you for the suggestion. Physician prescribed trandate 5 mg intravenously for the treatment of her high blood pressure at the emergency department. We have added it in the 2^nd^ paragraph on the page 6.

We used facial mask with 100% oxygen at the operating room, instead of a nasal cannula to improve her oxygen saturation. We have mention it in the 2^nd^ paragrap on page 7.

8-Why you decided to start with general anesthesia in spite of chest x-ray finding and low oxygen saturation

Response: Thank you for the suggestion. The patient could not lie flat on the table for surgery. Abdominal motion caused by rapid respiratory pattern would compromise the surgical manipulation under spinal anesthesia. We have revised and described more details of physical examination in the 2^nd^ paragraph on the page 6 and the 1^st^ paragraph on the page 7.

9- During operation Fluid input is mentioned but urine output not (fluid chart is needed)

Response: Thank you for the suggestion. We have revised and mention it in the 1^st^line on the page 8.

10- What is your explanation for decreased urine output in the 1st post Op day
Response: Since renal function was normal (BUN: 14.2 mg/dL, Cr: 0.64 mg/dL) check at 8^th^ hour after surgery and at admission, inadequate cardiac output was the cause of decreased urine output. We have revised and mention it on the page 8.

11-In 1st post operative day why you did not check for intraperitoneal blood loss or DIC. Also tachycardia is not a sign of cardiomyopathy with low LVEF (please explain)
Response: Thank you for the suggestion. Physical examination did not reveal distention of abdomen, vaginal bleeding, but distention of neck veins was found. Laboratory data did not show significant anemia. It may suggest the patient did not have hypovolemic status. Additionally, we could not find any condition disposed to DIC including massive blood loss and transfusion, disruption of operation wound and thrombocytopenia.

Tachycardia is not a sign of cardiomyopathy with low LVEF, but we cannot exclude heart failure in the presence of low cardiac output. Echocardiography is essential to any patient of undefined hypotension, and it is easy to perform in the ICU. More important, cardiac function evaluated by echocardiography can be a guide for the treatment of heart failure or hypovolemia associated with low cardiac output. We have mention the findings of echocardiography in the 1^st^ paragraph on the page 9.

12-Again elevated WBCS and C-reactive protein without elevated temp

Response: Thank you for the suggestion. The patient had just undergone surgical operation. The patient still had mild fever of 37.5°C besides elevated WBCS and C-reactive protein. Significant high fever was not found in the patient maybe resulted from early phase of infection.

13-Blood pressure totally not mentioned all through post operative days

Response: Thank you for the suggestion. The blood pressure was 150/90 on arrival in the ICU. For every 4 hours during intensive care, the BP was 140/90, 130/88/, 120/70. At the 14^th^ hour, her blood pressure declined to 90/60 mmHg. We have revised and mention it on the page 8.

14-no clinical or biochemical data from day 1 to day 4 post operative

Response: Thank you for the suggestion. We have revised and mention it on the page 8 and page 9.

15-Again no information about her cardiac state at discharge and what medications she received

Response: Thank you for the suggestion. Owing to the improvement of hemodynamic parameters, a marked resolution of pulmonary edema in the chest radiology, and activity corresponding to class II of the New York Heart Association (NYHA) Functional classification, she was discharged with the medication as that of the ward. However, we admit that there is a flaw in not repeating an echocardiogram before her discharge. Two years after discharge from the hospital, repeat echocardiography showed that her LVEF had recovered to 52%.

16-You diagnosed gestational hypertension depending on what (one measurement at admission)

Response: Thank you for the suggestion. Definition of gestation hypertension: High blood pressure that develops after week 20 in pregnancy and goes away after delivery.

17-Endomyocardial biopsy (EMB) has variable sensitivity & specificity and the risk-benefit ratio being high, why you decided to do EMB?.
Response: Thank you for the suggestion. The investigation of EMB, which is discussed and recommend in some literatures, had not performed in our case, even in our hospital.

18-Rapid diagnostic tests (RDT) for viral influenzas' often used earliest in clinical settings, why you did not use it early?

Response: Thank you for the suggestion. Prompt delivery of the fetus is best management of a parturient woman with high blood pressure and pulmonary edema. Since the symptoms and signs were not obvious at the ED, we did not have routine RDT test for a pregnancy woman at emergence department before COVID pandemic. In addition, the result of this test will not alter the supportive treatment of airway at that moment. However, we must admit that there is a flaw in the consideration of early diagnosis.

19- Do you think it was very rapid recovery and discharge within 7 days only?

Response: Thank you for the suggestion. Influenza- related PPCM is a rare disease, but most patients reported in the literature can recover if treated correctly. Early diagnosis and treatment with antiviral agent (oseltamivir) since postpartum day 2 were applied for this patient. So the patient can recover rapidly including pulmonary edema and restoration of hemodynamic parameters. Finally the patient was discharged based on steady daily activity and symptoms corresponding to New York Heart Association Functional Classification Class II. We believed that it was a safe discharge option.

20-The paragraph in paper 10 and 11(Owing to…….nullification of the antibody titer) totally contradictory, could not understand.

Response: Thank you for the suggestion. An elevated antibody indicated the patient had influenza infection. Antibody titer was negative (nullification) and recovery of cardiac function after the administration of immunoglobulin.

*Thanks for reviewer’s guidance. The whole manuscript had been revised based on the reviewer’s comments and suggestions. The authors pay very carefully to revise the manuscript and make sure every point from the reviewers have been checked and revised. Thanks for reviewer’s all useful and important comments. Those comments make this paper much better.*
